# Advancements in Therapeutic Deep Eutectic Solvents as Multifunctional Transdermal Delivery Systems

**DOI:** 10.3390/pharmaceutics18030360

**Published:** 2026-03-13

**Authors:** Ke Li, Bo Yan, Zhibo Cao, Rongrong Lu, Guotai Wu, Yang Hai

**Affiliations:** 1 Gansu Pharmaceutical Industry Innovation Research Institute, Gansu University of Chinese Medicine, Lanzhou 730101, China; ke.lee@outlook.com (K.L.);; 2 Key Laboratory of Chemistry and Quality, Gansu University of Chinese Medicines, Lanzhou 730101, China; 3 Research and Experimental Center, Gansu University of Chinese Medicine, Lanzhou 730101, China

**Keywords:** therapeutic deep eutectic solvents (TheDES), transdermal drug delivery, stratum corneum, eutectogels, lipid exchange

## Abstract

The human skin, specifically the stratum corneum (SC), remains a formidable barrier to the delivery of therapeutic agents. Nearly 80% of new drug candidates exhibit poor solubility or low permeability, necessitating the development of novel delivery vehicles. Therapeutic deep eutectic solvents (TheDES) have emerged as a “green,” multifunctional solution capable of significantly enhancing the solubility and transdermal flux of active pharmaceutical ingredients (APIs). This review discusses the physicochemical fundamentals of TheDES, mechanisms of permeation enhancement—including the newly identified “lipid exchange” effect—and the transition from liquid solvents to advanced dosage forms such as eutectogels and nanoemulsions. Current therapeutic applications in pain management, infectious diseases, and chronic conditions are highlighted, alongside critical assessments of dermatological safety and future directions in computational modeling.

## 1. Introduction

The transdermal delivery of therapeutic agents offers several clinical advantages, including avoidance of first-pass hepatic metabolism and the ability to maintain relatively stable plasma drug concentrations [1]. However, the stratum corneum (SC), often described by the “bricks and mortar” model (protein-rich corneocytes embedded in a lipid matrix), acts as the primary barrier to exogenous substances [2]. For effective passive diffusion, drug molecules typically require a molecular weight below 500 Da and an appropriate partition coefficient (log P) that enables sufficient lipid–water balance [3].

Conventional chemical permeation enhancers (CPEs), such as ethanol and surfactants, are often limited by skin irritation or systemic toxicity [4]. In recent years, deep eutectic solvents (DESs) and their therapeutic counterparts, therapeutic deep eutectic solvents (TheDES) have emerged as promising alternatives. Originally recognized in materials science as eutectic mixtures and in thermal engineering as phase-change materials, these systems have gained attention in green chemistry due to their tunable physicochemical properties and functional versatility [5,6]. Within the framework of green chemistry, these mixtures have evolved into what are now widely known as DES or low-transition temperature mixtures, reflecting their distinctive physicochemical properties and broad functional potential. These are liquid mixtures formed by hydrogen bond acceptors (HBAs) and donors (HBDs) that exhibit a significant melting point depression compared to their individual components [7].

From a formulation perspective, the relevance of eutectic systems becomes especially apparent when considered alongside the biopharmaceutical classification system, which categorizes drugs based on their solubility and permeability characteristics. Many compounds that fall into classes associated with limited solubility have been investigated as components of eutectic systems to improve dissolution and absorption profiles [8]. The integration of active pharmaceutical ingredients into DES frameworks thus represents a promising strategy for optimizing bioavailability while minimizing pharmacokinetic variability. The incorporation of active pharmaceutical ingredients (APIs) into DES frameworks has therefore become an attractive strategy for improving bioavailability while reducing pharmacokinetic variability. This concept has evolved further into TheDES, in which at least one component possesses therapeutic activity. In these systems, API can function either as hydrogen bond donors or acceptors, enabling the formation of supramolecular assemblies that integrate solvent and drug functions into a single phase. This approach offers significant potential to address long-standing challenges in drug delivery, particularly the poor aqueous solubility that affects a large proportion of therapeutic compounds and limits their bioavailability. By modifying intermolecular interactions at the molecular level, TheDES can enhance solubility, stabilize amorphous forms, and modulate drug release behavior. By incorporating the API directly into the eutectic network, TheDES serve as both the drug and the vehicle, providing a sustainable bridge between green chemistry and advanced biomedicine [9].

Overall, the evolution from conventional eutectic mixtures to DES and ultimately to TheDES reflects a shift from purely physicochemical curiosity toward purpose-driven pharmaceutical innovation. Understanding the molecular interactions, thermodynamic principles, and compositional flexibility that govern these systems provides the essential foundation for their development as advanced transdermal delivery platforms, where both stability and biological compatibility must be precisely balanced to achieve therapeutic effectiveness.

## 2. Design and Physicochemical Fundamentals

During the formation of DES, an extensive hydrogen-bond network is established, supported by van der Waals interactions and electrostatic repulsion, which collectively contribute to a depression of the melting point, allowing the mixture to remain in the liquid state at ambient temperature [10]. A mixture is classified as “deep eutectic” when the observed freezing point depression exceeds that of an ideal liquid mixture, indicating strong negative deviations from ideality [11]. DES are generally composed of an HBA and HBD that interact through a complex network of noncovalent forces, including hydrogen bonding, dipole–dipole interactions, van der Waals forces, and, in some cases, halogen bonding [12]. These interactions collectively disrupt the crystalline lattices of the individual components, resulting in significant melting point depression and the formation of a stable liquid phase at or near ambient temperature. The design of TheDES centers on establishing a robust supramolecular hydrogen bond network between the HBA and HBD [13]. The structural versatility of DES is often described using generalized formulations that account for ionic and molecular constituents, emphasizing the modularity that enables tailored physicochemical properties. This tunability has facilitated the extension of DES applications from industrial and environmental contexts into biomedical and pharmaceutical fields. 

### 2.1. Selection of HBA/HBD for Skin Compatibility

The design of skin-compatible TheDES is critical for achieving efficient transdermal delivery while maintaining dermatological safety. Unlike industrial solvents that primarily emphasize dissolution performance, component screening for transdermal systems must be conducted within a multidimensional evaluation framework that balances permeation enhancement capability, biodegradability, cytotoxicity, and long-term skin tolerance. The goal is to facilitate drug transport across the SC without causing irreversible disruption of barrier function.

Component selection must therefore consider compatibility with the biochemical composition of the skin barrier. The SC is composed primarily of ceramides, cholesterol, and free fatty acids arranged in highly ordered lamellar structures [14]. Ideally, TheDES components should induce only transient and reversible perturbations of this structure, enhancing permeability while allowing rapid barrier recovery. Such reversibility distinguishes well-designed TheDES from traditional chemical penetration enhancers that may cause prolonged irritation or barrier damage.

To improve safety profiles, DES components are often selected from compounds with established pharmaceutical or nutritional acceptance. Substances classified as Generally Recognized as Safe (GRAS) by the U.S. Food and Drug Administration (FDA), or those with established pharmaceutical applications, such as provitamins, amino acids, and approved excipients, are particularly desirable due to their favorable toxicological profiles. Choline chloride (ChCl) is among the most frequently used HBAs because of its low cost, biodegradability, and regulatory acceptance as a nutritional component in several regions [15]. Its quaternary ammonium structure enables the formation of stable hydrogen bond networks with diverse HBDs, resulting in significant melting point depression and enhanced physicochemical stability. Additionally, as shown in Table 1, choline geranate (CAGE) ionic liquid (IL)/DES may contribute to solubilization of hydrophobic and hydrophilic drugs and delivery of intractable drugs through physiological barriers by stabilizing proteins and nucleic acids [16]. Mitragotri’s group showed that CAGE can deliver large proteins safely. In porcine skin, CAGE enabled deep penetration of bovine serum albumin, ovalbumin and insulin without degrading their structure. In rats, topical CAGE/insulin induced a strong 40% drop in blood glucose (4 h post-dose) with sustained effect, implying effective protein transport and activity [17]. As research progresses, HBAs such as betaine are increasingly recognized for their superior dermatological compatibility and multifunctional benefits. Betaine, a naturally occurring zwitterionic compound that meets GRAS criteria, exhibits exceptional water-retention capacity and can mimic the action of natural moisturizing factors within the SC [18]. Belén Olivares et al. [19] investigate the use of betaine-ure DES as a drug delivery system to enhance the activity of the imipenem antibiotic. The research highlights that imipenem is preferentially solvated by betaine molecules within the DES, and demonstrates that betaine–urea DES exhibits non-toxicity towards skin cells. Song et al. [20] found TheDES based on betaine and ascorbic acid (AA) increases the solubility of AA in polyol solvents, enabling the preparation of highly concentrated AA topical formulations with minimal water. These properties suggest that DES formulations based on betaine can serve dual roles as both delivery vehicles and bioactive excipients, contributing to improved skin tolerance during repeated application.

The selection of HBDs is equally critical, as their molecular structures directly influence interactions with the SC lipid matrix. Medium- to long-chain fatty acids have emerged as particularly advantageous candidates due to their intrinsic permeation-enhancing properties [21]. Capric acid (decanoic acid, C10:0) has recently gained attention as a structurally determinant HBD owing to its natural origin and dual functionality in hydrogen bonding and membrane interaction [22]. With moderate lipophilicity, capric acid effectively partitions into intercellular lipid bilayers while maintaining sufficient molecular mobility. When incorporated into DES, capric acid disrupts lipid packing through a fluidization mechanism, temporarily reducing barrier density and facilitating drug diffusion. Importantly, this interaction is typically reversible, allowing the lipid matrix to reorganize once the solvent is removed. This reversibility distinguishes fatty acid-based DES from more aggressive chemical enhancers that may cause long-term barrier impairment. Other fatty acids, including lauric acid (C12:0) [23] and oleic acid (C18:1) [24], have demonstrated similar potential. Oleic acid is also the “kink” in its structure caused by a cis double bond which introduces significant steric hindrance into lipid bilayers, markedly increasing membrane fluidity [24]. However, the selection of fatty acids must carefully balance chain length, melting point, and viscosity. Excessively long carbon chains may lead to higher melting points and increased viscosity, reducing spread ability and limiting practical application, whereas shorter-chain fatty acids may exhibit higher volatility and acidity, potentially causing skin irritation. Consequently, fatty acids within the C8–C18 range are generally considered optimal for constructing skin-compatible DES, as they provide a suitable compromise between permeation enhancement and formulation stability.

Terpenes, such as menthol and borneol, are frequently employed as HBDs or co-solvents due to their strong permeation-enhancing effects [25]. Their mechanisms involve the disruption of lipid packing, increased lipid extraction, and facilitation of both intercellular and intracellular diffusion pathways [26,27]; despite their effectiveness, terpene-based systems require careful concentration optimization to minimize irritation risk. At elevated concentrations, terpenes may extract excessive lipids or induce cytotoxic effects in keratinocytes, leading to erythema or edema [28]. Studies indicate that the relationship between terpene concentration and skin safety is nonlinear, with concentrations typically within the 1–5% (*w*/*w*) range demonstrating favorable tolerance profiles. Therefore, component screening should incorporate not only physicochemical characterization but also dermatological evaluation metrics such as transepidermal water loss, skin impedance, and histopathological analysis to establish a safe therapeutic window.

Another emerging consideration in HBA/HBD selection is the role of intermolecular interaction strength in determining solvent performance. Strong hydrogen bonding interactions can enhance melting point depression and solubilization capacity but may also increase viscosity, potentially limiting diffusion and skin spreading. Conversely, weaker interactions may reduce viscosity but compromise system stability. Thus, achieving an optimal balance between hydrogen bond strength and molecular mobility is essential for maximizing transdermal efficiency. Recent studies suggest that introducing secondary interactions, such as van der Waals forces or π–π stacking, can provide additional flexibility in tuning solvent properties without compromising skin compatibility [24].

An advanced strategy in DES design involves the concept of drug–excipient integration, wherein the active pharmaceutical ingredient itself functions as a structural component of the eutectic system [29]. This approach represents a paradigm shift from traditional formulations, which rely on inert solvents constrained by drug solubility limits. By contrast, TheDES are formed through direct molecular interactions between APIs and complementary co-formers. For example, poorly soluble natural compounds such as paeonol can form eutectic systems with menthol or lactic acid, simultaneously improving solubility and leveraging synergistic pharmacological effects [30]. This strategy not only enhances drug loading capacity but also reduces the need for additional excipients, potentially minimizing toxicity and simplifying regulatory approval pathways.

In addition to molecular design considerations, future research should increasingly emphasize patient-centered formulation criteria, including sensory properties, ease of application, and long-term dermatological acceptability. Factors such as odor, residue, and evaporation rate may influence patient adherence, particularly in chronic treatment settings. Therefore, HBA/HBD screening should extend beyond laboratory performance metrics to include real-world usability considerations. Collectively, these perspectives highlight that the rational selection of DES components is not solely a chemical optimization process but a multidisciplinary challenge integrating pharmaceutical science, dermatology, and materials engineering.

### 2.2. Thermodynamic Stability and Eutectic Point

Thermodynamic stability represents a defining feature of TheDES, as the formation of eutectic systems often results in reduced crystallinity, depressed melting points, and enhanced resistance to environmental stressors. Encapsulation of drugs within TheDES matrices has been shown to improve stability against degradation factors such as light, temperature fluctuations, oxidative conditions, and pH variation, thereby extending the functional lifespan of labile therapeutic molecules [31]. The investigation of eutectic behavior and phase stability has been widely supported by analytical studies aimed at elucidating hydrogen bonding interactions, solid–liquid equilibria, and thermal transitions within these systems, where vibrational spectroscopy, Fourier-transform infrared (FT-IR) spectroscopy, nuclear magnetic resonance (NMR), Differential Scanning Calorimetry (DSC), and microscopic examination were applied [32]. Hydrogen bond formation, which underpins eutectic stability, has been extensively examined through spectroscopic and thermal analyses. For instance, the presence and strength of intermolecular hydrogen bonding have been investigated using FT-IR, where characteristic peak shifts provide evidence of molecular association. Complementary calorimetric studies have demonstrated that TheDES formation is typically accompanied by significant melting point depression and modified decomposition profiles. In one study, Ramya M. Subramani et al. [33] reported that hydrogen bond formation within a therapeutic eutectic system was verified through infrared spectral shifts, while thermal analysis revealed decomposition temperatures ranging from 200 to 400 °C, indicating altered stability compared to the individual α-hydroxy acid components.

Intermolecular interactions governing eutectic formation have also been investigated through nuclear magnetic resonance studies, which provide molecular-level insights into hydrogen bonding environments and component compatibility [34]. Ana Rita C. Duarte et al. demonstrated that eutectic formation between menthol and an active pharmaceutical ingredient was driven by specific hydrogen bond interactions, as evidenced by chemical shift variations observed in both proton and carbon spectra [35]. In related work, the interaction between decanoic acid and ketoconazole was shown to involve hydrogen bonding between the imidazole amine group and the carboxylic acid moiety, contributing to the observed melting point depression and enhanced liquid-phase stability at ambient conditions [36]. Thermal behavior associated with eutectic formation has been further explored through gravimetric and calorimetric studies, which collectively indicate that supramolecular interactions can influence volatility and degradation pathways. In particular, eutectic formation has been reported to moderately enhance the thermal stability of fatty acid-based systems, suggesting that hydrogen bond networks may reduce molecular mobility and delay decomposition processes [37].

Although less commonly applied, mass spectrometric investigations have recently provided new insights into the supramolecular organization of TheDES. Gas-phase studies have revealed the presence of stable molecular clusters that reflect strong intermolecular associations within eutectic systems. For example, Oluseyi Olawuyi et al. employed advanced mass spectrometric approaches to characterize cluster structures in menthol–ibuprofen eutectic mixtures, offering direct evidence of persistent hydrogen-bonded assemblies beyond the condensed phase [38].

Collectively, these findings highlight that the thermodynamic stability of TheDES is closely linked to the strength and organization of intermolecular interactions, which govern eutectic point depression, phase behavior, and resistance to environmental stress. A comprehensive understanding of these parameters is therefore essential for the rational design of stable and effective transdermal formulations.

### 2.3. Viscosity and Density

High viscosity is widely recognized as a common limitation of DES, as it can reduce mass transfer efficiency and limit diffusion rates within the system [39]. This stems from its highly ordered and dense internal hydrogen bond network structure, which significantly restricts the free movement of molecules [40]. Taking the classic choline chloride/urea (1:2) DES as an example, its viscosity at room temperature can reach several hundred mPa·s [41].

Nevertheless, viscosity can be effectively modulated without compromising eutectic integrity. Studies have shown that the introduction of small amounts of water or moderate increases in temperature can weaken intermolecular hydrogen bonding interactions, thereby improving fluidity while preserving the characteristic physicochemical properties of the DES [39]. Such tunability is particularly valuable in transdermal applications, where optimized viscosity is essential to ensure adequate spreading, skin contact, and controlled drug diffusion.

In addition to viscosity, DES typically exhibit relatively high density ranging from 1.1 to 1.4 g/cm^3^, values that exceed those of water and many conventional organic solvents [42,43]. From a formulation perspective, density also influences drug partitioning behavior and interfacial interactions with biological membranes.

In practical applications, viscosity and density must be considered synergistically. While high viscosity may hinder mass transfer and slow drug diffusion, elevated density can enhance phase stability and improve separation efficiency. Therefore, rational DES design requires a careful balance between these properties, achieved through appropriate selection of hydrogen bond donors and acceptors that maintain sufficient fluidity while preserving structural cohesion. Such optimization is particularly critical in transdermal delivery systems, where both rheological behavior and interfacial compatibility significantly influence therapeutic performance. Table 1 lists the physicochemical properties and characterization of some commonly used TheDES.

**Table 1:** Physicochemical properties and characterization of TheDES used for transdermal delivery systems.

HBA	HBD	Molar Ratio	Thermodynamic Stability	Skin Compatibility	Viscosity	Density	Toxicity	Refs.
Ascorbic acid	Betaine	1:1	No specific peak within −40–110 °C	Reduced levels of aging biomarkers and resulted in uniform skin tone	—	—	—	[20]
Ibuprofen/Benzoic acid/Phenylacetic acid	Menthol	1:3/1:3/1:2/1:1	Good stability in the range of 24.85–49.85 °C; poorer stability with ester formation observed in some systems Decompose in 108.12–165.03 °C	—	High/—/—/—	—	selectively kills cancer cells	[35,38,44]
Choline chloride	Malonic acid/Ethylene glycol/Lactic acid	1:1/1:2/1:3	High stability (no complete decomposition at 300 °C)/Low stability (complete decomposition at 300 °C)/Moderate stability (complete decomposition at 300 °C)	—	Highest viscosity (434.2 mPa·s/Intermediate/Lowest	—	Low toxicity, safe and biocompatible	[45]
Ketoconazole	Decanoic acid	1:5	Clear liquid maintained at room temperature for at least 5 months	—	415.64 mPa·s (25 °C)	—	—	[36]
Choline	Geranic acid	1:2	Changes minimally after exposure to heat or UV stress	Some skin changes were observed, but completely disappeared after a 14-day recovery period	56,919 mPa·s (25 °C)	0.989 ± 0.001 g/mL (25 °C)	Low, nearly negligible toxicity	[16,46]
Marine	Decanoic acid/Lauric acid/Myristic acid	1:1	—	Exhibits cytotoxicity	25 °C,362/349/356 mPa·s	1.03439/1.01961/1.00723 g/mL (25 °C)	Stronger antiproliferative activity and higher cytotoxicity than pure marine.	[37]
Menthol	Caprylic acid	80:20 (*w*/*w*)	90% curcumin retained after 30 days in HDES-based ME-23; stability comparable to HDES-based microemulsions	—	—	—	No relevant cytotoxicity in HaCaT cells, potentiated wound healing, and presented antibacterial properties	[25]
Tetrabutylammonium bromide	Polyethylene glycol 200	1:2.12/1:3.05/1:2.21/1:2.99/1:2.09/1:3.14	—	—	170.47/115.88/198.77/157.14/249.36/182.32 mPa·s	1.0976/1.0990/1.1054/1.1085/1.1108/1.1136 g/mL	—	[47]

### 2.4. Classification of TheDES in Transdermal Delivery

**Figure 1:**
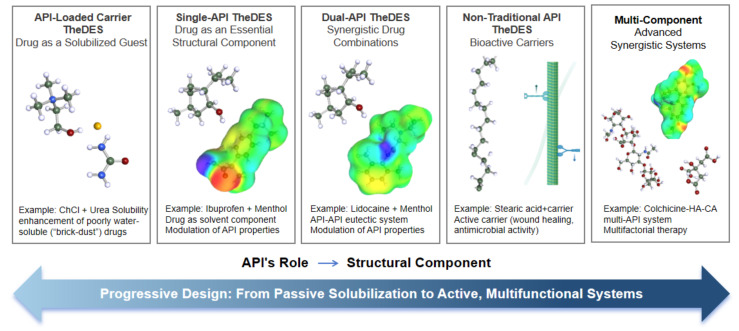
Overview of TheDES components and their classification.

DESs are conventionally categorized into several types based on the chemical nature of their constituents, such as combinations of quaternary ammonium salts with metal chlorides, organic acids, or other hydrogen bond donors. While this compositional framework provides a useful foundation for understanding DES formation, it does not fully capture the functional complexity of TheDES, particularly in transdermal drug delivery. Figure 1 provides an overview of TheDES components and their corresponding classification.

In the context of transdermal delivery, it is crucial to distinguish whether an API acts as a structural component of the eutectic network or as a solubilized guest molecule that becomes especially significant. This distinction directly influences key parameters such as thermodynamic stability, drug release kinetics, permeation enhancement, and biological compatibility with the skin barrier. Unlike traditional formulations where excipients and drugs serve clearly separated roles, TheDES often blur these boundaries by integrating therapeutic molecules into the solvent structure itself. As a result, the supramolecular organization of the system becomes inseparable from its pharmacological performance.

Based on the structural and functional relationship between the API and the eutectic framework, TheDES can be broadly classified into several categories that reflect a progressive design philosophy, ranging from systems in which the carrier dissolves the drug to those in which the drug itself becomes an integral component of the delivery matrix. This continuum highlights the evolution of formulation strategies from passive solubilization toward active, multifunctional systems capable of simultaneously enhancing solubility, permeability, and therapeutic synergy. Such a classification approach not only clarifies current research trends but also provides a conceptual framework for guiding the rational design of future TheDES tailored for transdermal applications.

Accordingly, TheDES used in transdermal delivery can be divided into five categories depending on whether the API functions as a required structural component of the eutectic system or exists as a solubilized entity within it. These categories illustrate a gradual transition from “vehicle-dissolved drug” systems to more advanced designs in which therapeutic molecules themselves serve as structural and functional building blocks of the eutectic matrix, thereby maximizing both physicochemical and biological performance.

#### 2.4.1. Single-API TheDES: Drug as an Essential Structural Component

Single-API TheDES refer to eutectic systems in which the API itself acts as a necessary structural component, functioning either as an HBA or HBD within the supramolecular network. In these formulations, the API directly participates in eutectic formation with a biocompatible conformer, leading to significant melting point depression and the generation of a homogeneous liquid or semi-solid phase at or near room temperature. This structural integration distinguishes single-API TheDES from conventional formulations, as the drug is no longer merely a solute but an integral part of the solvent architecture.

Such systems are particularly advantageous for transdermal delivery because they enable intrinsic modification of the drug’s physicochemical properties without chemical derivatization. For example, the ibuprofen–menthol eutectic system demonstrates how hydrogen bonding interactions can transform a crystalline API into a stable liquid state, thereby enhancing solubility, diffusivity, and skin permeation [35]. The resulting improvement in transdermal flux highlights the potential of this approach to overcome solubility limitations while maintaining therapeutic efficacy. Similarly, He, H et al. [21] prepared TheDES from the antidepressant mirtazapine (MTZ) and medium-chain fatty acids (e.g., decanoic acid, DA). They showed that the MTZ–DA TheDES was thermodynamically stable and greatly increased transdermal flux: the MTZ–DA formulation gave a cumulative 8 h skin penetration of ~98 µg/cm^2^ and a 6.4-fold increase in bioavailability over MTZ alone. In all of these examples, the drug is integral to the eutectic, and no inert carrier is present. Therefore, by embedding the API within the eutectic framework, single-API TheDES offer a rational strategy for simultaneously optimizing drug stability and delivery performance.

#### 2.4.2. Dual-API TheDES: Synergistic Drug Combinations

Dual-API TheDES are formed when two therapeutically active molecules interact through hydrogen bonding or other noncovalent forces to create a unified eutectic system. In these formulations, both components serve as functional elements of the supramolecular structure, often acting reciprocally as hydrogen bond donors and acceptors. This configuration not only reduces melting points and enhances solubility but also introduces the possibility of synergistic therapeutic effects, making such systems particularly suitable for localized and multifunctional treatments [31]. A representative example is the lidocaine–flurbiprofen (Lidocaine: Flurbiprofen, 1:1) eutectic system, in which the anesthetic and anti-inflammatory agents form a homogeneous phase characterized by a single glass transition temperature and significantly improved physicochemical properties compared to their individual forms [48,49]. This dual-drug integration facilitates enhanced skin permeation and localized therapeutic action while potentially minimizing systemic exposure. The design of dual-API TheDES underscores the potential of eutectic engineering to merge pharmacological synergy with physicochemical optimization, providing a promising platform for combination therapy in transdermal applications [32,49].

#### 2.4.3. API-Loaded Carrier TheDES: Drug as a Solubilized Guest

API-loaded carrier TheDES are characterized by eutectic systems formed from non-therapeutic or minimally active components, into which an API is subsequently incorporated as a dissolved solute. In this configuration, the drug does not directly participate in the hydrogen bond network but benefits from the unique solvent properties of the DES matrix. Such systems are especially effective for enhancing the solubility and stability of poorly water-soluble compounds, often referred to as “brick dust” drugs, thereby facilitating their transdermal transport.

A classical [50] example of this type involves inert or low-activity components such as ChCl and urea forming a “blank” DES carrier, which then solubilizes the API. For instance, L-arginine and 2-hydroxypropyl-β-cyclodextrin in a 4:1 ratio can form a series of DESs with varying water content, these DES systems significantly enhance the solubility of several poorly soluble drugs: ibuprofen, norfloxacin, and nateglinide solubility increased by 79.3-, 44.1-, and 3.2-fold, respectively, compared to aqueous solutions. In addition, these DES carriers effectively promote transdermal delivery of lidocaine, with permeation enhancement attributed to increased SC lipid fluidity and alterations in keratin structure [51].

The underlying mechanisms which improved permeation in the API-loaded carrier TheDES include the disruption of SC lipid organization, transient modification of protein conformation, and increased thermodynamic activity of the solubilized drug. The tunable viscosity and polarity of the carrier facilitate controlled release and prolonged skin contact, which are critical parameters for efficient transdermal delivery. Furthermore, these systems preserve the structural integrity of the API, minimizing degradation or crystallization while maximizing bioavailability.

Overall, “blank” DES carriers demonstrate considerable potential for high-efficiency loading and the delivery of poorly soluble drugs. Their versatility allows them to be tailored for specific APIs and therapeutic targets, bridging the gap between solubility enhancement and transdermal performance while maintaining skin compatibility. This approach exemplifies the practical utility of TheDES in pharmaceutical formulation, providing a flexible platform for both research and clinical application.

#### 2.4.4. Non-Traditional API TheDES: Bioactive Carriers with Intrinsic Therapeutic Function

Non-traditional API TheDES consist of eutectic systems formed from biologically active molecules that are not conventionally classified as pharmaceutical drugs but exhibit inherent therapeutic properties. In these formulations, the eutectic mixture itself serves as the active therapeutic entity rather than merely functioning as a delivery medium. Typically derived from natural compounds such as fatty acids, terpenes, or organic acids, these systems demonstrate that supramolecular interactions can generate bioactive formulations with multifunctional properties.

A representative example is the menthol–stearic acid (Menthol:SA, 8:1) [27]. In this system, menthol acts as a natural cooling and permeation-enhancing agent, while stearic acid is a naturally occurring long-chain saturated fatty acid. Although neither component is a conventional pharmaceutical API, the TheDES formed from their combination functions as a highly active therapeutic unit. In vitro studies using human immortalized keratinocytes (HaCaT) have shown that this TheDES promotes cell migration, increasing wound closure rates by nearly 40% relative to controls, demonstrating clear wound-healing activity. Concurrently, it exhibits antimicrobial activity against Gram-positive bacteria, including methicillin-resistant *Staphylococcus aureus* (MRSA), highlighting its dual-functionality.

The therapeutic mechanisms of non-traditional TheDES are multifaceted. Menthol can transiently disrupt lipid packing in the SC and modulate keratinocyte signaling pathways, enhancing drug permeation and tissue regeneration. Long-chain fatty acids such as stearic acid contribute to lipid membrane interaction and provide structural support, while also exhibiting mild antimicrobial and anti-inflammatory effects. The eutectic interaction between components can also stabilize the liquid state, increase thermodynamic activity, and improve skin compatibility, creating a carrier system that is inherently bioactive and multifunctional.

This class of TheDES exemplifies a paradigm shift in transdermal formulation design, where the distinction between carrier and therapeutic agent is increasingly blurred. By integrating permeation enhancement, antimicrobial activity, and wound-healing potential into a single formulation, non-traditional API TheDES offer a versatile platform for biomedical applications, particularly in topical and transdermal therapies where multifunctionality and biocompatibility are critical. These systems can serve as active excipients or standalone therapeutics, reducing the need for additional drugs while maintaining safety and efficacy, which may streamline regulatory approval and formulation development.

#### 2.4.5. Multi-Component TheDES: Advanced Synergistic Therapeutic Systems

Multi-component TheDES represent an advanced design strategy in which three or more active molecules interact within a single eutectic framework to form a homogeneous, bioactive therapeutic system. This approach extends the concept of dual-API TheDES by integrating multiple pharmacologically active agents into one liquid-phase formulation, aiming to address complex or multifactorial pathological conditions through synergistic action.

A representative example is the colchicine (CO)–4-hydroxyacetophenone (HA)–protocatechuic acid (CA) ternary TheDES (molar ratio 1:1:1), developed for the management of rheumatoid arthritis [52]. All three components are derived from traditional medicinal sources, where CO serves as the primary anti-inflammatory and analgesic agent, while HA and CA provide skin permeation enhancement and adjunctive therapeutic effects. The eutectic mixture exhibits a significantly reduced melting point of 27.33 °C, facilitating its incorporation into transdermal patch formulations. In vivo studies demonstrated marked synergistic effects: the 24 h cumulative transdermal fluxes of CO, HA, and CA reached 32.26, 117.67, and 56.79 µg/cm^2^, respectively, with analgesic and anti-inflammatory outcomes exceeding those of commercially available diclofenac patches.

Mechanistically, the extensive hydrogen bonding network formed among multiple APIs plays a key role in enhancing transdermal delivery. For instance, CO alkoxy groups form hydrogen bonds with HA carbonyls, while CO amide groups interact with CA carboxyls, creating a dense supramolecular network. This network effectively “shields” the APIs from non-specific interactions with SC lipids, reducing drug retention within the epidermis and increasing flux across the skin. Additionally, the eutectic interaction enhances molecular mobility, suppresses crystallization, and maintains the liquid state at physiological temperatures, thereby improving thermodynamic activity and diffusion driving forces.

The multifunctionality of multi-component API TheDES extends beyond permeation enhancement. By integrating multiple therapeutic mechanisms within a single formulation, these systems enable synchronized drug release, reduced dosing frequency, and potential synergistic pharmacological effects, all of which are highly desirable for chronic inflammatory conditions or diseases requiring combination therapy. This approach highlights the evolution of TheDES from simple solubilization vehicles into sophisticated therapeutic platforms that combine rational molecular design with clinical functionality.

Future developments in this area may involve tailoring multi-component TheDES for personalized medicine, optimizing component ratios based on patient-specific pharmacokinetics, or incorporating functional excipients to further enhance stability, skin compatibility, or targeted delivery. Such strategies emphasize the versatility and translational potential of multi-component API TheDES in advanced transdermal therapeutics.

## 3. Mechanisms of Transdermal Permeation Enhancement

### 3.1. Lipid Bilayer Fluidization

**Figure 2:**
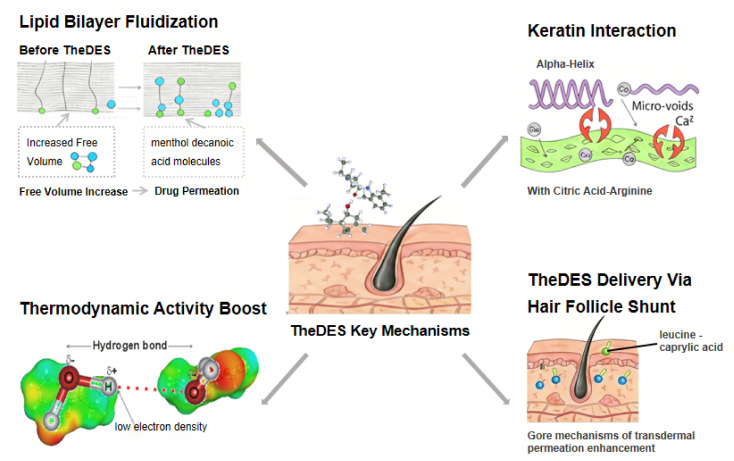
Schematic representation of the four core mechanisms of the stratum corneum (SC) barrier.

Unlike conventional permeation enhancers that typically rely on a single disruption pathway, as Figure 2 shows, TheDES operate via coordinated interactions with both lipid and protein components of the SC while simultaneously modulating drug thermodynamic activity. These combined effects enable more efficient and controlled drug transport across the skin barrier.

TheDES components insert into the tightly packed intercellular lipid lamellae of the SC, disrupting its order and increasing fluidity. For example, ATR-FTIR on excised human SC shows that hydrophilic DES mixtures (e.g., glycerol-based eutectics) shift the CH_2_ stretching peaks, indicating reduced lipid order, while preserving keratin’s α-helical content [53]. Similarly, synchrotron X-ray scattering experiments demonstrate that even small amounts of terpene–acid DES significantly decrease the lamellar repeat spacing under physiologic hydration [54]. In essence, TheDES alkyl chains intercalate among SC lipids, weakening van der Waals forces and hydrogen bonds. Unlike aggressive solvents such as ethanol or DMSO, which tend to solubilize and extract lipids outright, TheDES (being amphiphilic) allow tunable disruption: their composition can be adjusted to control polarity and viscosity [54,55]. Hydrophobic components commonly present in TheDES, such as menthol and medium-chain fatty acids, are capable of intercalating into the intercellular lipid matrix of the SC. This insertion disrupts the highly ordered arrangement of ceramides, cholesterol, and free fatty acids, creating structural irregularities or “kinks” that increase the free volume available for drug diffusion. At the molecular level, the alkyl chains of these hydrophobic constituents penetrate the lipid bilayers and weaken van der Waals interactions and hydrogen bonding between adjacent lipid molecules [27]. As a result, the lipid domains transition from a tightly packed, crystalline state to a more fluid and disordered configuration, thereby reducing diffusion resistance. Notably, ATR-FTIR studies also report that certain DES actually stabilize keratin simultaneously with lipid fluidization, suggesting a reversible membrane modulation rather than permanent extraction [53].

### 3.2. Keratin Interaction

In addition to lipid interactions, TheDES can directly affect the protein structure of the SC by interacting with keratin filaments within corneocytes. These interactions may induce conformational transitions in keratin from an α-helix to a β-sheet structure, leading to partial disruption of the intracellular protein network. Such structural modifications reduce the mechanical integrity of the SC and may generate microcavities or transient aqueous channels that facilitate drug transport [54,55]. For instance, citric acid–arginine-based TheDES have been shown to interact electrostatically with keratin, promoting conformational rearrangement while chelating calcium ions that stabilize the protein matrix. Similarly, betaine–ascorbic acid systems can induce keratin modulation while simultaneously providing antioxidant protection, thereby minimizing potential oxidative stress associated with barrier perturbation [20,56].

### 3.3. Increased Thermodynamic Activity

Another important mechanism is the enhancement of drug thermodynamic activity. By maintaining APIs in an amorphous or liquid state, TheDES increase the chemical potential of the drug and thus the driving force for diffusion across the skin. According to thermodynamic principles, drug flux is proportional to its activity, expressed as a = γc, where γ is the activity coefficient and c is the concentration. Hydrogen-bond interactions within TheDES inhibit drug crystallization, stabilizing the API in a high-energy amorphous form in which the activity coefficient approaches unity. This state significantly enhances diffusion potential compared to crystalline forms. This drives stronger diffusion through the skin. Quantitatively, a menthol–fatty acid TheDES dissolved risperidone ~7300-fold better than water [27]. Another study [21] reported similarly dramatic solubility boosts. In practice, this means that a TheDES formulation can maintain a high concentration gradient at the skin surface. For example, in vitro studies of a mirtazapine–decanoic acid TheDES found the ATR-FTIR spectrum of treated SC had significantly altered-CH_2_ bands, consistent with enhanced drug flux. Although full molecular dynamics data are still emerging, these observations collectively imply that TheDES-loaded APIs exert a much greater driving force than crystalline drugs or simple organic solvents. The effect is compounded by DES viscosity: many TheDES form viscous liquids that cling to skin, prolonging contact and sustaining the concentration gradient for hours.

### 3.4. Lipid Exchange Effect

Unlike short-chain solvents that leach out lipids en masse, some TheDES appear to engage in a controlled lipid exchange process. TheDES possess Hansen solubility parameters that closely match those of human skin lipids, enabling them to partially integrate into or reorganize the lipid matrix without causing irreversible barrier damage. Through this process, TheDES may form a transient, solvent-rich microenvironment within the SC that enhances drug partitioning and diffusion [54]. For instance, leucine–caprylic acid systems exhibit strong compatibility with skin lipids and can penetrate into deeper layers while maintaining barrier integrity. In certain cases, this lipid exchange process may also contribute to barrier recovery by promoting lipid reorganization after drug delivery [56], making TheDES particularly suitable for applications involving compromised or sensitive skin [57]. 

Overall, TheDES act on multiple SC targets simultaneously. They fluidize intercellular lipids (shown by FTIR and X-ray studies), mildly modulate keratin (with preserved cell structure), and maintain drugs in a high-activity state [53,54]. Many of these mechanistic insights come from human SC ex vivo or animal-skin models. For instance, ATR-FTIR experiments were performed on human epidermis samples, whereas other studies used hairless mouse or porcine skin to visualize structural changes. In every case, TheDES outperformed conventional enhancers: they produce comparable (or higher) permeation flux with far less lasting disruption of the skin. Thus, TheDES leverage a synergistic, multi-pronged enhancement strategy that contrasts with the one-dimensional action of single-mode enhancers like ethanol or DMSO.

## 4. Advanced TheDES-Based Dosage Forms

**Figure 3:**
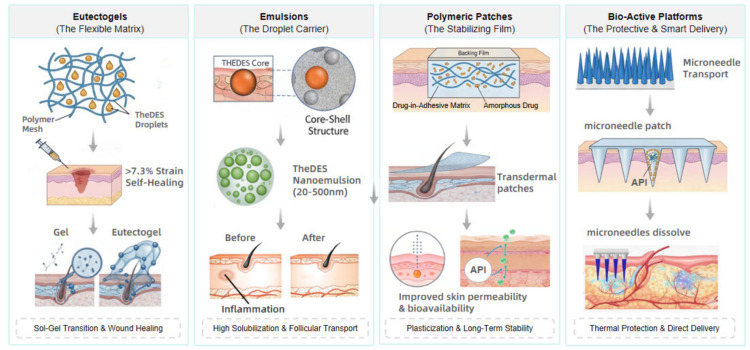
Representative therapeutic deep eutectic system (TheDES)-based dosage forms and their structural characterization, delivery mechanisms, and therapeutic and transdermal applications.

The immobilization of liquid TheDES into solid or semi-solid platforms is essential for clinical utility. The corresponding dosage forms and their pharmaceutical applications are summarized in Figure 3 and Table 2.

### 4.1. Eutectogels (Supramolecular and Polymeric)

Eutectogels combine the properties of DES with the structural integrity of polymeric networks [15].

Co-gel systems based on TheDES are semi-solid, three-dimensional network structures formed by the physical or chemical integration of TheDES with supramolecular gelators or polymer networks. This type of formulation ingeniously combines the superior drug solubilization and permeation capabilities of TheDES with the adhesiveness, sustained-release properties, and patient compliance of gel systems, representing a cutting-edge strategy in the field of transdermal drug delivery. Their formation mechanisms can be primarily classified into two categories: supramolecular co-gels based on non-covalent interactions and polymer co-gels based on covalent or strong physical crosslinking, each with distinct focuses in terms of structure, performance, and application [58].

Gels are a class of widely used soft materials whose three-dimensional network structure can be formed by various gelators, such as polymers, inorganic nanoparticles, or small molecules (i.e., low-molecular-weight gelators, LMWGs) [59]. Gel-based DESs are called eutectogels [57]. Commonly used polymeric network materials include Carbopol, chitosan, sodium alginate, hydroxypropyl methylcellulose (HPMC), and polyvinyl alcohol (PVA). These polymers provide mechanical strength, dispersibility, and tunable viscosity to the gels. The preparation methods include direct mixing, water-DES hybrid gelation, in situ gelation, thermosensitive gelation, and nanogel formation. DESs form hydrogen-bonded solvent networks, while polymers construct three-dimensional gel matrices. After the combination of the two, the gel strength is enhanced, the microstructure is modified, the molecular dispersibility is improved, and physicochemical stability is strengthened. Compared with traditional gels, DES-based gels generally have higher viscosity, better elasticity, and a more compact internal network structure [60]. A self-healable and injectable polymeric eutectogel responsive to intracellular pH and temperature stimuli was developed through the interaction between imidazolium-based DESs and polyvinyl alcohol [61]. Fabricated via the interaction between imidazolium-based DES and polyvinyl alcohol, this gel serves as a local drug delivery system for cancer therapy, releasing the anticancer drug 5-fluorouracil to targeted sites under the intracellular pH 5.0 environment. It features a hierarchical porous 3D network structure woven by thick fibers, exhibiting high mechanical strength (>7.3% strain) as well as key properties including swelling ability, injectability and self-healing capability. In addition, the gel boasts excellent biocompatibility with a hemolysis rate below 2%, ensuring blood compatibility while maintaining the sustained viability of HaCaT cells.

Olles J R and colleagues [62] developed co-gels that rely on the self-assembly of TheDES with low-molecular-weight gelators through non-covalent interactions—including hydrogen bonds, van der Waals forces, and π–π stacking—to form a three-dimensional network that immobilizes the fluid TheDES. This gelation process is typically thermoreversible, meaning a sol–gel transition occurs upon heating and the gel state is recovered upon cooling, facilitating processing and filling. Parsana N and colleagues [63] have designed and prepared supramolecular eutectogels with simultaneous self-healing, injectable and ionic conductive properties using DES as the medium. In this study, the pharmaceutically active cetylpyridinium chloride (C_16_PyCl) and cetylpyridinium bromide (C_16_PyBr) were dissolved in DES to obtain the target gels. These eutectogels not only exhibit excellent self-healing ability, injectability, ionic conductivity and antimicrobial performance, but also possess high encapsulation efficiency for curcumin. The sustained-release behavior and release kinetics of curcumin from the gels were also investigated.

In some study [64], an efficient three-dimensional network was constructed via the entanglement of polymer chains, thereby preparing entangled eutectogels. The fabrication adopted a simple heating–cooling method, in which ultra-high molecular weight polyvinylpyrrolidone (PVP) was directly dissolved in the DES (reline). When the mass fraction of PVP was 40%, the as-prepared gel exhibited outstanding stretchability with a fracture strain of up to 1410%, a toughness of 544.8 kJ/m^3^, and an ionic conductivity of 0.015 S/m. Meanwhile, it could generate stable and reliable resistance signals, making it suitable for strain sensing applications. TheDES plays a dual role here: it acts not only as an excellent solvent for drug dissolution but also, through its own hydrogen-bond network, serves as a “co-assembly driving force” that participates in and reinforces the stability of the supramolecular network, such as lidocaine + lauric acid [65], decanoic acid + sodium laurate [66], and menthol + lauric acid [59].

Chitosan-based TheDES hydrogels possess a nanostructured network, where the particle size of spherical aggregates ranges from 20 to 50 nm. These aggregates can interact with amino and hydroxyl groups, thereby enhancing the material’s biocompatibility, biodegradability, and thermal stability (thermal stability ranges: 50–120 °C and 130–200 °C). Both the biological activity and substance extraction efficiency are significantly improved [67].

### 4.2. TheDES-Based Nanoemulsions and Microemulsions

The limited aqueous solubility of many APIs remains a major challenge in pharmaceutical development, often restricting bioavailability and therapeutic efficacy. To address this issue, increasing attention has been directed toward advanced drug delivery systems capable of enhancing solubility and permeability while maintaining drug stability [31]. Nanoemulsions and microemulsions are emerging drug delivery systems that have gained significant attention in pharmaceutical development. These systems exhibit excellent characteristics such as transparency and thermodynamic stability, making them suitable for the delivery of both hydrophilic and hydrophobic drugs [68].

Nanoemulsions (NEs) are heterogeneous systems composed of two immiscible liquids stabilized by emulsifiers or surfactants. Due to their excellent drug delivery properties, they have shown great potential in medical applications. The droplet size of nanoemulsions ranges from 20 to 500 nm, and they represent a non-homogeneous system in which one liquid (dispersed phase) is dispersed in the form of nanoscale droplets within another liquid (continuous phase) [69]. NEs protect encapsulated compounds from degradation and enhance barrier crossing [70]. Using TheDES as the internal phase can increase the solubility of poorly soluble drugs (e.g., docetaxel) by up to 20-fold [24]. A self-emulsifying drug delivery system (SEDDS) was prepared using a (caprylic acid: cannabidiol, 3:1) TheDES as the oil phase, which, upon dispersion in water, formed uniform nanoscale droplets. Under strongly acidic gastric conditions (pH 1.2), the system effectively protected cannabidiol for up to 2 h and demonstrated the ability to permeate Caco-2 cell monolayers with ease [71]. Moreover, Jayoung Kim et al. [72] prepared stable nanocomplexes with particle sizes under 100 nm by formulating a TheDES composed of choline bicarbonate and oleic acid in a 2:1 ratio, combined with the small-molecule drug Vitepofin. These nanocomplexes efficiently overcome biological barriers, including cellular uptake and retention, penetration of 4T1 tumor spheroids, and accumulation in tumor tissue in vivo. Saif Syed et al. [73] prepared a nanoemulsion based on a TheDES composed of thujaplicin and valproic acid (1:1). The formulation exhibited an antioxidant EC_50_ of 44.76 ± 0.99 μg/mL and a cytotoxic IC_50_ of 174.43 ± 23.49 μg/mL, and was able to induce chromatin condensation and nuclear fragmentation (hallmarks of apoptosis) in cancer cells. Scratch wound-healing assays further demonstrated that the formulation inhibited HeLa cell migration.

Microemulsions (MEs) are characterized by very small droplet sizes, typically ranging from 10 to 100 nm, and outperform other delivery carriers in both transdermal and subcutaneous administration. They are particularly suitable for lipophilic compounds, effectively solubilizing drugs and enhancing their bioavailability. Their application in dermal delivery offers several advantages, including improved skin permeability, increased drug stability [74,75], and controlled drug release kinetics [68,76]. A microemulsion prepared using a lidocaine–ibuprofen deep eutectic mixture as the oil phase enhanced the transdermal delivery of artemisinin [77]. Jayoung Kim et al. [78] developed a TheDES composed of choline and rosmarinic acid (1:2), which, when formulated with apomorphine, self-emulsified into a microemulsion upon subcutaneous injection, thereby slowing apomorphine release and prolonging its pharmacokinetic profile. Tianxiang Yin et al. [79] prepared an oil-in-water microemulsion using a TheDES based on psoralen and paeonol (2:8) as the oil phase, exhibiting superior aqueous solubility and bioactivity compared to the pure active pharmaceutical ingredients. Collectively, these studies demonstrate that TheDES-based microemulsions provide enhanced bioavailability of active pharmaceutical ingredients. Moreover, Jieyu Wu et al. [65] reported that a TheDES composed of caffeic acid and lidocaine (1:1) was able to effectively form non-aqueous microemulsions without the need for additional surfactants. With increasing 1,2-propylene glycol (PG) content, the system underwent a structural transition from water-in-oil (W/O) to oil-in-water (O/W) via a bicontinuous phase, and within a specific water content range, it functioned as a gelling agent to form a gel.

### 4.3. Polymeric Patch Matrices

#### 4.3.1. Plasticizers

TheDES can act as plasticizers for polymers like Eudragit or HPMC, increasing film flexibility and preventing drug recrystallization [43]. For example, a rotigotine-lactic acid TheDES patch remained crystal-free for six months due to stabilization of the amorphous state.

#### 4.3.2. Penetration Enhancers

TheDES composed of oxymatrine and fatty acids (e.g., lauric acid) has been investigated as a novel transdermal penetration enhancer. Its inherent components can synergistically disrupt the SC structure, thereby significantly improving the transdermal delivery efficiency of drugs. Active moieties within TheDES (e.g., the carboxyl groups of lauric acid and the N^+^-O^−^ groups of oxymatrine) exert intermolecular interactions to synergistically perturb the ordered lipid arrangement of the skin SC, which consequently enhances the transdermal permeation efficiency of both the TheDES-loaded active ingredients and other incorporated drugs [80].

#### 4.3.3. Crosslinking Agents

TheDES can dynamically modulate the network structure through hydrogen bonding interactions with polymer chains, thereby regulating drug release profiles. For example [81], vanillin, acting as a natural crosslinking agent, forms reversible Schiff–base bonds (dynamic covalent bonds) via its aldehyde groups with the amino groups of chitosan; simultaneously, its phenolic hydroxyl groups establish multiple hydrogen bonds with the hydroxyl or amino groups on polymer chains. This dual action of “dynamic covalent bonds + hydrogen bonds” constructs a dense network with self-healing properties, which can significantly retard the diffusion of hydrophilic drugs and achieve sustained drug release. This mechanism is analogous to the crosslinking of gelatin or chitosan by tannic acid, where the abundant phenolic hydroxyl groups of tannic acid bind to polymers via strong hydrogen bonding to form physical crosslinking sites, regulating network density and stability, and further controlling drug release kinetics. Therefore, through such dynamic and reversible non-covalent interactions, TheDES enables precise modulation of polymer network structures to realize on-demand design of drug release behaviors.

#### 4.3.4. Stabilizers and Antioxidants

TheDES can form a protective hydrogen-bonded network and utilize its intrinsic antioxidant components to improve the chemical stability of labile drugs in formulations. For example, Olivares et al. [82] demonstrated that imipenem, a β-lactam antibiotic, exhibits higher stability in a betaine/urea (1:1.5) mixture than in aqueous solutions; similarly, Lee et al. [83] confirmed that human interferon α2 retains its structural integrity and biological activity after long-term storage at 37 °C in a choline chloride/fructose (1:1) mixture. From a transdermal perspective, such stabilization is highly relevant, as many dermally delivered drugs—particularly peptides, anti-inflammatory agents, and photosensitive compounds—are prone to hydrolysis, oxidation, or recrystallization. Incorporation into TheDES may help maintain drugs in a molecularly dispersed state, enhance thermal stability at skin temperature, and provide additional antioxidant protection when redox-active components are present. However, despite encouraging evidence from bulk DES systems, systematic evaluation of TheDES as stabilizing matrices in transdermal formulations remains limited. This underexplored aspect represents a promising direction for future research, particularly for oxidation-sensitive and thermolabile drugs intended for dermal delivery.

**Table 2:** TheDES-integrated dosage forms and their reported pharmaceutical applications.

Dosage Form Type	Classification/Core Composition	Preparation Mechanism	Performance Characteristics	Application Examples	References
Eutectogels	Polymeric Eutectogels Core: TheDES + polymers (Carbopol, chitosan, PVA, ultra-high molecular weight PVP, etc.)	1. Conventional: Mixing/gelation; covalent/strong physical crosslinking for 3D network. 2. Entangled (polymeric subcategory): Heating–cooling; 3D network via polymer chain entanglement.	1. Conventional: High mechanical strength, adjustable viscosity, responsiveness, biocompatibility (hemolysis rate <2%). 2. Entangled: Excellent stretchability (fracture strain 1410%), toughness, stable ionic conductivity.	1. Conventional: Imidazolium DES-PVA gel for 5-fluorouracil release; chitosan-based hydrogel (20–50 nm). 2. Entangled: Ultra-high molecular weight PVP-reline gel for strain sensing.	[67,84,85]
	Supramolecular Eutectogels Core: TheDES + low-molecular-weight gelators (LMWGs, e.g., C_16_PyCl, C_16_PyBr)	Noncovalent self-assembly; thermoreversible sol–gel transition (heating-sol, cooling-gel).	Self-healing, injectable, antimicrobial, high drug encapsulation efficiency, easy processing.	NADES-based gel for curcumin-sustained release; menthol-lauric acid TheDES supramolecular gel.	[59,61]
	Polymeric Eutectogel-based Microneedles Core: TheDES + polymers Polymerizable DES (PDES)	Polymer crosslinking solidification; PDES-based rapid fabrication.	High stretchability (>1600%), strong skin adhesion (>30 kPa), excellent mechanical strength.	Advanced microneedle patches for transdermal drug delivery with enhanced adherence.	[61]
	ion-gel	Self-polymerization of HEMA and 3D ion-gel network formation.	Stable properties and rapid drug release.	ChCl: Ascorbic acid (2:1) +HEMA	[67,86]
Emulsions (Nano- and Microemulsions)	Nanoemulsions (NEs) Structure: TheDES as the internal phase, droplet size 20–500 nm.	Stabilized by emulsifiers; TheDES-based SEDDS form nanodroplets upon contact with water.	Acts as a stabilizer, enhancing antimicrobial activity, bioavailability, and drug distribution.	PRF: NAC(6:4), Caprylic acid: Cannabidiol (3:1), DL-menthol: thymol (2:8), Thymol:Raspberry ketone (9:1)	[71,85,87,88]
	Microemulsions (MEs) Structure: TheDES as the oil phase, droplet size 10–100 nm.	Surfactant-free non-aqueous microemulsions; structural transitions occur with variations in 1,2-propylene glycol content.	Enhances solubilization; acts as a stabilizer; improves bioactivity and pharmacological performance; and prolongs the pharmacokinetic profile	Choline: Geranic acid (1:2), Lidocaine: Ibuprofen (1:1), Osthole: Paeonol (2:8), Tetrabutylammonium chloride: Decanoic acid (1:2), Lactic acid: Menthol (2:1)	[74,75,77,78,79,89]
Polymeric Patch Matrices (Drug-in-Adhesive)	Plasticizer Target: Polymers (Eudragit, HPMC)	Blended with polymers to improve flexibility and inhibit drug recrystallization.	Enhances film flexibility, stabilizes drug amorphous state.	Rotigotine-lactic acid TheDES patch, maintaining crystal-free state for 6 months.	[24]
	Penetration Enhancer Core: Oxymatrine + fatty acids (e.g., lauric acid)	Synergistically disrupts SC lipid structure via intermolecular interactions.	Markedly improves transdermal permeation efficiency of drugs.	Oxymatrine-lauric acid TheDES, boosting transdermal absorption of itself and other drugs.	[69]
	Crosslinking Agent Mechanism: Dynamic covalent bonds + hydrogen bonds	Crosslinks polymers (e.g., chitosan) to form dense self-healing network; similar to tannic acid crosslinking.	Regulates polymer network, achieves hydrophilic drug sustained release.	Vanillin-chitosan crosslinked network; tannic acid crosslinked gelatin/chitosan, controlling drug release kinetics.	[58,90]
	Stabilizer and Antioxidant Mechanism: Protective hydrogen bond network + antioxidants	Forms stable system with unstable drugs via hydrogen bond network.	Enhances drug chemical stability, maintains structure and activity.	1. Betaine/urea (1:1.5) TheDES improves imipenem stability. 2. ChCl/fructose (1:1) TheDES preserves interferon α2 activity at 37 °C.	[60,90]

## 5. Therapeutic Applications

### 5.1. Pain Management

**Figure 4:**
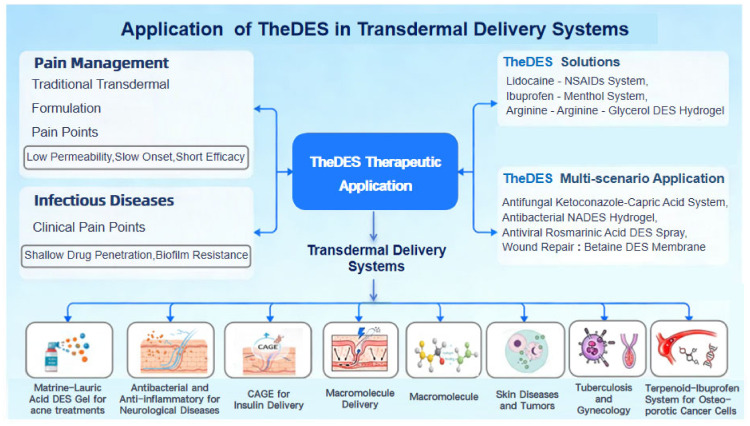
Application scenarios, core advantages, and technical characteristics of therapeutic hydrophobic deep eutectic solvent-based transdermal drug delivery systems.

Pain is one of the most common clinical symptoms, which can be divided into acute pain (e.g., postoperative pain, traumatic pain) and chronic pain (e.g., neuropathic pain, cancer pain). Uncontrolled persistent pain can seriously impair the quality of life of patients. As illustrated in Figure 4, the application scenarios, principal advantages, and technical features of TheDES-based transdermal systems are summarized.

Transdermal drug delivery systems have become an important administration route for pain management owing to their advantages, such as avoiding the hepatic first-pass effect associated with oral administration, maintaining stable plasma drug concentration, and reducing systemic adverse reactions. Traditional transdermal analgesic formulations (e.g., patches, gels) are often plagued by bottlenecks including low drug skin permeation efficiency, slow onset of action, and relatively short duration of efficacy [91]. In contrast, TheDES, as multifunctional transdermal delivery carriers, can break through the limitations of the skin barrier through their intrinsic properties, providing novel solutions for the efficient delivery of analgesic drugs.

Lidocaine-based TheDES with anti-inflammatory drugs (NSAIDs, ketoprofen, flurbiprofen) show significantly reduced melting points and enhanced local analgesia [92]. An ibuprofen–menthol system demonstrated a 12-fold increase in drug solubility and a three-fold increase in permeability [35]. A study [91] developed an arginine–glycerol (Arg:Gly) DES system, which increased the solubility of ibuprofen by 7917-fold, with no cytotoxicity and enhanced anti-inflammatory activity. When this DES system was incorporated into an alginate-based hydrogel, the resulting gel exhibited a more homogeneous structure. The cumulative release rate of ibuprofen reached 93.5% within 8 h, and the transdermal permeation amount was 8.5 times higher than that of the ibuprofen-only hydrogel, providing an efficient strategy for the transdermal delivery of NSAIDs.

### 5.2. Infectious Diseases

DES have attracted increasing attention in the treatment of infectious diseases due to their molecular design flexibility, biocompatibility, and intrinsic antimicrobial properties. Infectious skin diseases, including fungal infections and bacteria-associated wounds, present two major challenges: insufficient drug penetration into deeper tissue layers and biofilm formation that contributes to antimicrobial resistance. TheDES provide innovative solutions by combining solubilization, permeation enhancement, and synergistic antimicrobial effects.

For example, a ketoconazole–capric acid TheDES achieved a 6.2-fold higher transdermal flux compared with commercial formulations and effectively inhibited Candida albicans biofilm formation. Polyvinyl alcohol (PVA)/chitosan hydrogels based on DES have also been shown to accelerate the healing of methicillin-resistant Staphylococcus aureus (MRSA)-infected wounds by reducing inflammation and promoting collagen deposition.

Bioactive DES composed of natural compounds have demonstrated promising results in pathogen inhibition and tissue repair [93]. A rosmarinic acid–proanthocyanidin–glycol DES incorporated into a PVA–borate hydrogel spray exhibited strong binding affinity to monkeypox virus proteins through hydrogen bonding and van der Waals interactions, significantly suppressing viral activity. Under acidic wound conditions (pH 5.5), the cumulative drug release reached 84.83%, while the formulation provided multifunctional benefits, including antibacterial, antioxidant, hemostatic, and UV-protective effects.

In bacterial wound healing, DES systems have further expanded therapeutic possibilities through synergistic strategies [94]. A betaine–levulinic acid DES served as a solubilizing carrier for curcumin and was incorporated into pullulan-based films, significantly improving curcumin’s water solubility and photostability [95]. The resulting dressing demonstrated adhesive strength four times greater than commercial hydrogels and sustained drug release. In ex vivo skin models, MRSA bacterial loads were reduced to below detectable levels, while fibroblast migration and re-epithelialization were promoted. DES formulations containing betaine, glucose, and glycol have also been used to stabilize silver, copper, and selenium nanoparticles, showing broad-spectrum antimicrobial and antiviral activities, thereby offering potential for treating multi-pathogen infections. These findings collectively demonstrate that DES-based systems, through structural modification and formulation integration, can effectively address challenges such as poor drug solubility, limited targeting capability, and delayed wound healing, providing strong technical support for clinical interventions in infectious diseases.

### 5.3. Chronic Conditions

Owing to unique physicochemical properties and biocompatibility, TheDES demonstrate irreplaceable advantages in the field of drug delivery. They are facile to prepare, cost-effective, biodegradable, and highly tunable, offering a distinctive and highly attractive platform for the delivery of therapeutics for such diseases [31,96]. Research on using TheDES to improve the physicochemical properties of active pharmaceutical ingredients is continuously expanding.

In the field of anti-bacteria, TheDES prepared from matrine and lauric acid (3:7) exhibited excellent solubilization capacity for curcumin, with curcumin demonstrating high stability in the DES solution. In water, the TheDES functions as a gelling agent to form a deep eutectic gel, displaying notable antioxidant activity as well as inhibitory effects against *Cutibacterium acnes* [59]. Joana M. Silva et al. [97] revealed the antibacterial potential of fatty acids in the form of DES. In the decanoic acid–myristic acid (CA:MA) system, significant antibacterial activity was observed against the tested Gram-positive bacteria and *Candida albicans*, with the decanoic acid–lauric acid (CA:LA) system exhibiting the strongest overall inhibitory (bactericidal) activity [97].

In neurological disorders, a SEDDS composed of TheDES and apomorphine prolonged the drug’s half-life, optimizing the existing thrice-daily dosing regimen to an alternate-day schedule and thereby improving patient compliance [78].

TheDES have also demonstrated potential in the delivery of macromolecules. CAGE has emerged as an effective carrier for large biomolecules, successfully delivering peptides such as insulin (~5.8 kDa) and siRNA into the dermis without compromising bioactivity. Similarly, a SEDDS containing apomorphine extended the drug’s half-life, allowing dosing frequency to be reduced and improving patient compliance [16].

In inflammatory and dermatological conditions, Menghan Li et al. [98] developed TheDES composed of menthol and fatty acids (decanoic acid, caprylic acid, oleic acid), all of which enhanced the anti-inflammatory activity of curcuminoids and curcumin. Among these, the oleic acid–menthol (1:1) system exhibited excellent biocompatibility and strong anti-inflammatory activity, significantly enhancing the anti-inflammatory effects of curcumin while reducing its cytotoxicity. The combination of oleic acid, menthol, and curcumin completely inhibited NO production without causing notable cytotoxicity. Moreover, CAGE has been demonstrated to have therapeutic potential in the treatment of rosacea. A clinical study involving 26 patients with mild to moderate facial rosacea showed that treatment with a CAGE-based gel significantly reduced the number of inflammatory lesions [46].

In oncology research, researchers have formulated TheDES using terpenes such as menthol, linalool, and saffron aldehyde in combination with the nonsteroidal NSAID ibuprofen. Studies demonstrated that saffron aldehyde: ibuprofen (3:1 and 4:1) and menthol: ibuprofen (3:1) exhibit significant therapeutic activity against colorectal cancer cells, displaying selective cytotoxicity toward cancer cells while enhancing ibuprofen permeability and solubility of [99]. Similarly, TheDES based on paclitaxel show synergistic effects and selective activity against colorectal cancer cells. The bioactivity of TheDES is molar-ratio-dependent, improving both antibacterial and anticancer properties, with perillene:ibuprofen (3:1) identified as the most effective system for inhibiting HT29 cell proliferation without compromising normal cell viability [100]. Additionally, TheDES composed of natural terpenes (carvacrol, menthol) and ibuprofen exhibited cytotoxicity against the HT29 cancer cell model, demonstrating the potential to reduce cancer cell proliferation. Notably, menthol–ibuprofen (3:1) showed lower cytotoxicity toward normal colon cells while maintaining higher cytotoxicity against cancer cells [101].

In tuberculosis treatment, a formulation based on a TheDES composed of citric acid and L-arginine (1:1) was investigated for tuberculosis treatment. The TheDES was encapsulated using a gas-saturated solution microparticle technique with supercritical CO_2_ and the porosity of the microparticles facilitated the rapid release of TheDES while exhibiting no cytotoxicity toward L929 cells [102]. Similarly, Joana Gonçalves et al. [103] synthesized three TheDES of ethambutol using trisaccharose, glucose, and glycerol as starting materials, encapsulated within nanostructured lipid carriers. This strategy enhanced the stability of the TheDES, protecting ethambutol from degradation while preventing interactions with other anti-tuberculosis drugs. Additionally, converting ethambutol from its crystalline salt form into a deep eutectic liquid improved its solubility and permeability, thereby enhancing its therapeutic efficacy. Encapsulation of TheDES for tuberculosis treatment thus holds promise as a novel approach for clinical applications.

Finally, in gynecological disorders, a metronidazole–maleic acid TheDES demonstrated controlled drug release for up to seven days in simulated vaginal fluid, offering an effective alternative to oral therapy and conventional vaginal formulations for bacterial vaginosis treatment [104].

Collectively, these studies highlight the versatility of TheDES as multifunctional delivery platforms capable of addressing diverse therapeutic challenges across a wide range of chronic conditions.

## 6. Biocompatibility and Dermatological Safety

TheDES have shown significant potential for enhancing drug solubility and permeability, ultimately improving bioavailability. While TheDES are often “well-tolerated,” toxicity is highly system-specific [23]. Owing to the unique composition and properties of TheDES, their degradation products may break down into multiple species, some of which could exhibit toxicological profiles distinct from those of the parent compounds. Furthermore, in certain systems, the enhanced permeation effect may disrupt the orderly and tight arrangement of keratinocytes or induce keratin denaturation, leading to alterations in skin structure—effects that have not yet been thoroughly investigated [24,105].

### 6.1. TheDES as Biocompatible Drug Carriers

TheDES have been increasingly investigated as multifunctional drug carriers due to their ability to enhance solubility, membrane transport, and the controlled release of APIs. Studies [7] have found that when APIs are administered in the form of TheDES, their solubility, intestinal absorption, controlled release, and effective transmembrane transport are significantly improved. Researchers developed an innovative, non-cytotoxic ionogel carrier based on a natural TheDES composed of ascorbic acid and choline chloride (1:2), and designed a biocompatible malic acid–sunitinib-targeted release system. The study demonstrated that the DES system was non-toxic to HN-5 cells, and that the drug formulation within the ionogel remained stable for up to six months without any detectable degradation [106]. In addition, CAGE has been demonstrated to be a highly efficient drug delivery carrier for the transdermal, buccal, and subcutaneous administration of poorly soluble drugs, exhibiting extremely low toxicity, high skin permeability, and minimal irritation, while enabling targeted therapeutic effects with minimal side effects [16]. Zhiyuan Zhao et al. utilized a TheDES prepared from biocompatible and non-toxic amino acids (AA) and citric acid (CA) to mediate the transdermal delivery of mesoporous silica nanoparticles, achieving for the first time non-invasive transdermal delivery into the bloodstream [107]. A tandospirone-loaded transdermal patch based on a tandospirone–levulinic acid (1:3) TheDES system demonstrated increased drug loading capacity, improved permeation across the skin, prolonged systemic retention, and enhanced bioavailability compared with conventional formulation [108].

### 6.2. TheDES as Permeation Enhancers

One of the most notable advantages of TheDES is their capacity to transiently modulate biological barriers [109]; CAGE-based systems have been shown to act as reversible skin permeation enhancers by interacting with lipid domains in the SC. It is proposed that these systems can intercalate within intercellular lipid lamellae and intracellular domains, generating temporary microstructural openings that facilitate drug transport through both intercellular and transcellular pathways [55].

### 6.3. Toxicological Considerations

The toxicity of TheDES has been evaluated using in vitro cell cultures, animal models, and limited clinical observations. Although many TheDES exhibit low toxicity, their biological effects cannot be generalized and must be assessed individually [105].

Eutectic formation may alter cellular uptake and charge distribution, potentially increasing toxicity compared to that of individual components [15]. For example, a xylitol–choline chloride mixture showed higher cytotoxicity than pure xylitol in PrestoBlue and crystal violet assays [110]. Similarly, TheDES composed of matrine and fatty acids demonstrated enhanced antibacterial activity alongside increased anti-proliferative effects and cytotoxicity [37].

Even when individual components are classified as GRAS, eutectic formulations may be treated as NCEs due to altered physicochemical behavior. Consequently, regulatory approval may require compliance with ICH guidelines concerning impurities (Q3D) and residual solvents (Q3C) [111].

### 6.4. Skin Barrier Disruption

Although TheDES are primarily designed to enhance transdermal drug delivery, their interaction with skin structures may also lead to temporary or, in some cases, prolonged perturbation of the skin barrier. The permeation-enhancing capability of TheDES is largely attributed to their ability to fluidize SC lipids, disrupt hydrogen bonding networks, and alter keratin conformation [28,112,113]. While these effects facilitate drug diffusion, excessive lipid extraction or protein denaturation may compromise barrier integrity.

For instance, a microemulsion system using a lidocaine–ibuprofen TheDES as the oil phase enhanced the transdermal delivery of artemisinin but was observed to disrupt the ordered lipid arrangement of the SC, reducing barrier function and altering the surface morphology of skin [77]. These findings highlight the need for long-term dermatological safety assessments.

Importantly, barrier disruption induced by TheDES is often described as reversible, but systematic long-term studies remain scarce. Prolonged or repeated application could potentially lead to increased trans epidermal water loss (TEWL), skin sensitivity, or susceptibility to external irritants [114,115]. Therefore, future investigations should prioritize quantitative assessments of barrier recovery kinetics, lipid reorganization processes, and cumulative exposure effects to better define safe therapeutic windows.

Transdermal delivery of therapeutic molecules is frequently constrained by the intrinsic barrier properties of the skin, with the SC serving as the principal obstacle to permeation. A menthol–thymol-based TheDES was developed as a model system; two enhancement strategies were comparatively evaluated: (i) chemical permeation enhancement through the application of DES, which modify SC structure, and (ii) mechanical disruption of the skin barrier using microneedle pretreatment. The skin permeation rates of drugs delivered via DES alone, microneedle treatment alone, and a combination of both methods were systematically compared with those achieved using a conventional control vehicle [116]. The results demonstrated that both strategies were capable of compromising the barrier function of the skin; however, the magnitude of permeation enhancement was strongly correlated with the lipophilicity of the drug molecule. Lipophilic compounds generally exhibited greater permeation improvement, suggesting that drug–vehicle affinity and partitioning behavior play decisive roles in transdermal delivery efficiency.

Further mechanistic insights were provided by the work of Mina Sakuragi et al. [117], who investigated choline chloride–glycerol-based DES systems. Their findings revealed that both neat and hydrated DES formulations can selectively extract lipids from the short lamellar phases of the SC, thereby compromising structural integrity. This extraction process disrupts the orthorhombic packing arrangement of lipid hydrocarbon chains, leading to increased membrane fluidity and reduced diffusional resistance. Such structural perturbations at the molecular level are considered a primary mechanism by which DES enhance transdermal drug transport, highlighting their potential as versatile chemical permeation enhancers in advanced topical and transdermal therapeutic systems.

### 6.5. Effects on Wound Healing

DES- and TheDES-based materials have shown promise in wound management due to their intrinsic antibacterial activity, anti-inflammatory potential and cytocompatibility [118]. These properties enable multifunctional therapeutic effects, including infection control, moisture retention, and support of tissue regeneration.

Nanofiber [118] and hydrogel [119] systems incorporating TheDES have shown the ability to reduce microbial load while promoting fibroblast proliferation and collagen deposition, suggesting advantages for chronic wound treatment. Beyond serving as passive carriers, certain TheDES components—such as organic acids, amino acids, and natural terpenes—may actively modulate the wound microenvironment by influencing pH, oxidative stress, and cytokine expression. However, the impact of TheDES on wound healing is highly dependent on formulation parameters. Excessive permeation-enhancing effects or local dehydration caused by certain hydrophobic DES systems could potentially delay epithelialization. Furthermore, while in vitro cytocompatibility results are generally favorable, in vivo responses may vary depending on wound type, duration of exposure, and the immune status of the patient.

Thus, future research should aim to clarify the balance between antimicrobial efficacy and tissue regeneration, with particular attention to inflammatory signaling pathways, angiogenesis, and scar formation. Long-term in vivo studies and standardized evaluation protocols will be essential to validate the clinical safety of TheDES-based wound therapies.

Silva, J.M. [27] and co-workers investigated deep eutectic systems composed of menthol and saturated fatty acids with varying alkyl chain lengths. Among the formulations studied, the menthol–stearic acid eutectic system exhibited the strongest hydrogen-bonding interactions, which contributed to enhanced physicochemical stability. Importantly, this system demonstrated negligible cytotoxicity and showed a pronounced ability to accelerate wound healing. In vitro experiments further confirmed that the eutectic solvent significantly promoted the migration of human immortalized keratinocytes (HaCaT cells), highlighting its potential as a bioactive platform for skin regeneration and topical therapeutic applications.

In addition to biochemical and structural factors, the intrinsic electrical properties of the skin have increasingly been recognized as critical regulators of wound healing. Endogenous electric fields generated at injury sites play a pivotal role in directing cell migration, proliferation, and tissue repair. However, in chronic wounds such as burns, diabetic ulcers, and pressure sores, the intensity of these endogenous electric fields is significantly reduced compared to that in acute wounds, which partly explains delayed healing outcomes. Xinkai Li et al. [120] developed a conductive double-network deep eutectic gel by polymerizing a polymerizable deep eutectic solvent (PDES) composed of acrylamide, choline chloride, and glycerol, followed by crosslinking with thiolated hyaluronic acid through disulfide bond formation. Their study demonstrated that, when combined with exogenous electrical stimulation, this conductive eutectic hydrogel effectively reduced inflammatory responses, enhanced cell proliferation and migration, accelerated collagen deposition, and promoted angiogenesis, ultimately facilitating skin tissue remodeling [121].

Conventional hydrogel dressings primarily function by maintaining a moist wound environment and preventing infection, rather than actively modulating cellular behavior. In contrast, electrical stimulation has been shown to directly influence skin cell dynamics, thereby accelerating wound closure. Ting Huang et al. [119] developed a multifunctional conductive hydrogel based on a glucose–choline chloride DES platform. Their findings revealed that the synergistic application of this hydrogel with electrical stimulation significantly enhanced hair follicle regeneration, angiogenesis, and collagen deposition, leading to improved wound healing efficiency. These advances underscore the expanding role of DES-based materials not only as passive delivery vehicles but also as active participants in bioelectrical and regenerative therapeutic strategies [119].

### 6.6. Skin Irritation Studies

Experimental studies indicate that certain TheDES exhibit minimal irritation potential. For example, a decanoic acid–menthol system significantly enhanced drug solubility while showing no signs of edema or inflammation in acute dermal irritation tests conducted on albino rabbits, suggesting good dermatological compatibility [122].

Despite these encouraging findings, irritation responses cannot be generalized across all TheDES formulations. Hydrophobic components, organic acids, and high hydrogen-bond donor concentrations may increase the risk of irritation by altering skin pH or disrupting lipid homeostasis. Moreover, synergistic interactions between components may lead to unexpected biological effects that are not predictable from individual constituents alone.

Another important consideration is the distinction between acute and chronic exposure. While short-term studies often report low irritation potential, repeated application could trigger cumulative irritation, sensitization, or subclinical inflammation. Therefore, standardized dermatological testing protocols, including human patch tests and biomarker-based inflammation assessments, are necessary to establish comprehensive safety profiles.

Overall, the dermatological compatibility of TheDES should be evaluated using a case-by-case approach, integrating physicochemical characterization with biological response data to ensure both efficacy and safety.

## 7. Future Directions and Conclusions

### 7.1. Future Directions

A significant bottleneck in TheDES development is the reliance on empirical trial-and-error for component selection. Recent advances in Artificial Intelligence (AI) and Machine Learning (ML) are addressing this by leveraging models such as Deep Neural Networks (DNN) and Random Forest to forecast drug entrapment with up to 93.0% accuracy. Future efforts will focus on training these algorithms on large molecular datasets to predict not only solubility but also skin kinetics, potentially reducing the need for costly and time-consuming in vivo permeation tests. Furthermore, in silico tools like COSMO-RS (Conductor-like Screening Model for Real Solvents) have been used to screen large libraries of eutectic mixtures, drastically boosting solubility outcomes. In one study, COSMO-RS screened 870 natural DES candidates for methotrexate, identifying combinations that increased solubility by over 600-fold [123]. These approaches highlight the growing role of in silico methods in guiding solvent selection, optimizing molar ratios, and forecasting intermolecular interactions. In addition to COSMO-RS, hybrid approaches combining molecular dynamics (MD) and machine learning are emerging. For instance, integrated MD/COSMO simulations of thymol–caprylic acid DESs for pesticide extraction revealed strong H-bond networks and predicted high selectivity [31]. Collectively, such in silico methods from COSMO-RS to data-driven models are expected to guide rational TheDES formulation, optimizing component ratios and anticipating interactions before synthesis.

Beyond computational design, future research is expected to focus on the “smart” delivery formats that provide controlled or stimuli-responsive release (e.g., thermally or pH-sensitive networks) [124], such as stimuli-responsive hydrogels, microneedle systems, and bioadhesive patches. Such hybrid systems could enable controlled, on-demand drug release while minimizing skin irritation and systemic exposure. In particular, the combination of TheDES with polymeric networks to form eutectogels offers opportunities to tailor mechanical properties, enhance stability, and improve patient compliance. Microneedle patches are another exciting hybrid platform. Eutectogel-based microneedles (TheDES + photopolymerizable polymer) offer both strength and high drug loading. Insulin-loaded nanoparticles in these needles were rapidly delivered—the needles dissolved in ~3–8 min in mouse skin, creating microchannels and releasing insulin with good control. Engineering teams have also developed 3D-printed eutectogel microneedles with customizable geometries for tailored release profiles.

Another important direction involves expanding the scope of deliverable therapeutics. While most current studies focus on small-molecule drugs, increasing attention is being directed toward the transdermal delivery of macromolecules, including peptides, nucleic acids, and vaccines. Understanding the mechanisms by which TheDES interact with biological membranes and modulate protein stability will be critical for translating these applications into clinical practice.

A critical challenge for TheDES is safety and regulatory acceptance. Many TheDES components (e.g., choline chloride, organic acids, menthol) are GRAS individually, but their mixtures can have emergent behaviors. Rigorous toxicity screening is therefore essential. Notably, published reviews [105] highlight that TheDES appear generally well-tolerated in cell and animal studies, with low acute toxicity. For example, CAGE was found to have “minimal toxicity to epithelial cells as well as skin” in animal models. In fact, CAGE enhanced antibiotic delivery by >16-fold without causing irritation [125]. These positive results suggest some TheDES may be safely used on skin. Nevertheless, there are concerns about skin barrier compatibility. TheDES often work by disrupting SC structure. Such disruption could potentially compromise barrier function or cause irritation with repeated use. Thus, each new TheDES formulation needs dedicated dermal safety testing while current data are limited. Experts agree that long-term toxicity, degradant profiles, and clinical-safety evaluations remain sparse for most TheDES. On the regulatory front, TheDES lack a formal category, so approval routes are unclear. Complex approval processes and the compatibility of TheDES components with existing excipient guidelines have been cited as obstacles. The broad consensus is that standardized guidelines should be developed. This might include systematic in vitro panels (cytotoxicity, irritation) followed by targeted animal studies, tailored to the intended application. Regulatory authorities (FDA, EMA) will likely treat TheDES as novel excipient mixtures; transparency about each component’s status and any unique toxicological data will be critical. In summary, while TheDES hold promise as “greener” and multifunctional solvents, their novel compositions demand careful biocompatibility and regulatory scrutiny before clinical translation.

Furthermore, sustainability considerations are expected to shape future TheDES research. The development of bio-based, biodegradable, and environmentally benign components aligns with the broader principles of green chemistry and may enhance the translational appeal of these systems.

### 7.2. Conclusions

TheDES represent a paradigm shift in transdermal drug delivery, moving beyond conventional solvent roles toward multifunctional platforms that combine solubilization, permeation enhancement, and intrinsic therapeutic activity. By modulating intermolecular interactions, thermodynamic properties, and biological interfaces, TheDES offer unprecedented flexibility in addressing the challenges associated with poorly soluble and poorly permeable drugs.

The successful incorporation of TheDES into delivery formats such as eutectogels, hydrogels, and transdermal patches demonstrates their potential to improve drug stability, bioavailability, and patient adherence. Moreover, their ability to facilitate the transport of diverse therapeutic agents—including anti-inflammatory drugs, antimicrobials, hormones, and macromolecules—underscores their broad applicability across clinical fields.

Despite these advances, several challenges remain, including the need for systematic safety evaluation, long-term dermatological studies, and regulatory harmonization. Addressing these issues through interdisciplinary collaboration among chemists, pharmacologists, material scientists, and clinicians will be crucial for translating laboratory findings into practical therapeutic solutions.

Overall, continued innovation in TheDES design, supported by computational modeling, advanced formulation strategies, and comprehensive biocompatibility assessments, is expected to further expand their role in next-generation transdermal therapies.

## Data Availability

No new data were created or analyzed in this study.
